# Amiodarone Provides Long-Lasting Local Anesthesia and Analgesia in Open-State Mouse Nociceptors

**DOI:** 10.3389/fphar.2022.872477

**Published:** 2022-03-18

**Authors:** Masakazu Kotoda, Toru Matsuoka, Keiichi Wada, Selwyn Jayakar, Hirofumi Ino, Koji Kawago, Yasutomo Kumakura

**Affiliations:** ^1^ Department of Anesthesiology, Faculty of Medicine, University of Yamanashi, Kofu, Japan; ^2^ F. M. Kirby Neurobiology, Boston Children’s Hospital, and Department of Neurology, Harvard Medical School, Boston, MA, United States; ^3^ Department of Surgery Ⅱ, Faculty of Medicine, University of Yamanashi, Kofu, Japan

**Keywords:** amiodarone, anesthetics, inflammation, pain, TRPV1 channel

## Abstract

Local anesthetics with long-lasting effects and selectivity for nociceptors have been sought over the past decades. In this study, we investigated whether amiodarone, a multiple channel blocker, provides long-lasting local anesthesia and whether adding a TRPV1 channel activator selectively prolongs sensory anesthetic effects without prolonging motor blockade. Additionally, we examined whether amiodarone provides long-lasting analgesic effects against inflammatory pain without TRPV1 channel activator co-administration. In the sciatic nerve block model, 32 adult C57BL/6J mice received either bupivacaine, amiodarone with or without capsaicin (a TRPV1 agonist), or vehicle *via* peri-sciatic nerve injection. Sensory and motor blockade were assessed either by pinprick and toe spread tests, respectively. In another set of 16 mice, inflammatory pain was induced in the hind paw by zymosan injection, followed by administration of either amiodarone or vehicle. Mechanical and thermal sensitivity and paw thickness were assessed using the von Frey and Hargreaves tests, respectively. The possible cardiovascular and neurological side effects of local amiodarone injection were assessed in another set of 12 mice. In the sciatic nerve block model, amiodarone produced robust anesthesia, and the co-administration of TRPV1 agonist capsaicin prolonged the duration of sensory blockade, but not that of motor blockade [complete sensory block duration: 195.0 ± 9.8 min vs. 28.8 ± 1.3 min, F (2, 21) = 317.6, *p* < 0.01, complete motor block duration: 27.5 ± 1.6 min vs. 21.3 ± 2.3 min, F (2, 22) = 11.1, *p* = 0.0695]. In the zymosan-induced inflammatory pain model, low-dose amiodarone was effective in reversing the mechanical and thermal hypersensitivity not requiring capsaicin co-administration [50% withdrawal threshold at 8 h (g): 0.85 ± 0.09 vs. 0.25 ± 0.08, *p* < 0.01, withdrawal latency at 4 h (s) 8.5 ± 0.5 vs. 5.7 ± 1.4, *p* < 0.05]. Low-dose amiodarone did not affect zymosan-induced paw inflammation. Local amiodarone did not cause cardiovascular or central nervous system side effects. Amiodarone may have the potential to be a long-acting and nociceptor-selective local anesthetic and analgesic method acting over open-state large-pore channels.

## Introduction

Local anesthetics play significant roles in various clinical settings, including the management of perioperative and chronic pain (Lirk et al., 2018). However, currently available local anesthetics cannot entirely fulfill the demand for long-lasting anesthesia and analgesia (Roberson et al., 2011). Recent developments in regional anesthetic techniques combined with relatively long-acting local anesthetics allow local anesthesia up to several hours. However, this duration is insufficient to cover the entire perioperative pain period, and repetitive injections or catheter placement for continuous administration is often required (Paladini et al., 2020). Furthermore, more frequent or repetitive administration is common for the management of patients with chronic pain, which significantly lowers the patients’ quality of life and level of satisfaction.

Lidocaine has been used clinically for >70 years (Gordh, 2010). Bupivacaine and its enantiomer levobupivacaine are also widely used as more potent and longer-acting local anesthetics, with higher protein binding ratios and partition coefficient values than those of lidocaine 95% and 560 vs. 64% and 110, respectively (Taylor and McLeod, 2020). Although amiodarone is not typically classified as a sodium channel blocker, it reportedly exhibits lidocaine-like inhibitory activity on voltage-gated sodium channels (Kodama et al., 1997). The protein binding ratio of amiodarone is similar to or higher than that of bupivacaine (96%), and its partition coefficient value is remarkably high (> 100,000) (Chatelain et al., 1986; Ho et al., 2018), suggesting its potential as a potent and long-acting local anesthetic or as a lead compound for novel local anesthetics (Taylor and McLeod, 2020).

Another important requirement for anesthetic drugs is the selectivity for sensory neurons or nociceptors (Lirk et al., 2018; Jayakar et al., 2021). Growing research has focused on developing anesthetics that eliminate pain without hampering normal motor function. Positively charged sodium channel blockers are emerging as novel anesthetics that selectively block the nociceptors (Dib-Hajj et al., 2009; Tochitsky et al., 2021). These compounds cannot permeate the cell membrane and thus cannot bind to the intracellular binding sites of voltage-gated sodium channels and thus inhibit sodium currents. However, positively charged sodium channel blockers can selectively enter cells that express open-state large-pore channels and block sodium channels (Binshtok et al., 2007; Nakagawa and Hiura, 2013). Considering that amiodarone has a high pKa value (8.7) (Chatelain et al., 1986) and exists mostly in its cationic form in the tissues, this drug may be able to dominantly enter nociceptors when the cells exclusively present open-state large-pore channels, such as TRPV1 channels (Binshtok et al., 2007).

In this study, we used a mouse model of sciatic nerve block to test the hypothesis that amiodarone provides long-lasting local anesthesia and analgesia and that adding the TRPV1 channel activator capsaicin selectively prolongs its sensory anesthetic effects without affecting motor function. We also examined whether low-dose amiodarone alone could exert long-lasting analgesic effects against inflammatory pain in case of inflammation-activated large-pore channels, without necessitating a TRPV1 channel activator.

## Materials and Methods

All experiments were conducted in accordance with the guidelines of the National Institutes of Health and the International Association for the Study of Pain. The experimental protocol was reviewed and approved by the University of Yamanashi Animal Care Committee. All neurobehavioral assessments were conducted by individuals blinded to the study design and grouping.

### Animals

Adult C57BL/6J mice (age: 8–10 weeks; weight: 20–25 g) were purchased from Japan SLC (Tokyo, Japan). The mice were group-housed at 23 ± 2°C under a 12 h light–dark cycle with food and water available ad libitum. All experiments were performed under normal room light and at 23 ± 2°C.

### Drugs

Bupivacaine, capsaicin, and zymosan (Fujifilm Wako Pure Chemical, Osaka, Japan) were dissolved in 5% DMSO and 5% Tween20 in normal saline. Amiodarone (TOA EIYO, Tokyo, Japan) was dissolved in 80°C water and chilled to 20°C, and then diluted with normal saline with 5% DMSO and 5% Tween20. All drugs were freshly prepared before use.

### Experiment 1

#### Peri-Sciatic Nerve Injection (Sciatic Nerve Block Model)

A total of 32 adult C57BL/6J mice were randomly assigned to the following four groups (*n* = 8 each, four females and four males): 1% bupivacaine, 1% amiodarone with or without capsaicin (1 μg/μL), and vehicle (normal saline with 5% DMSO and 5% Tween20). Under isoflurane anesthesia and aseptic conditions, a 5-mm skin incision was made in the left upper thigh using a No. 11 surgical blade. Under the microscope, the sciatic nerve was identified through the fascia between the biceps femoris and gluteal muscles, and 20 µL of the drug solution was injected into the peri-sciatic region using a 30-gauge insulin needle such that the solution surrounded the nerve. Based on body surface area (BSA), 20 µL for a 25 g C57BL/6J mouse (BSA: 82.6 cm^2^) is considered equivalent to approximately 4 ml for a 60 kg adult human (BSA: 1.6 mm^2^) (Reagan-Shaw et al., 2008; Cheung et al., 2009). The skin incision was closed using a single suture of 5–0 nylon. Sensory and motor blockades were assessed using the pinprick and toe spread tests at baseline, 5, 10, 20, 30 min, 1, 2, 3, 4, 6, 8, and 12 h after drug injection.

#### Pinprick Test

The sensory block after the peri-sciatic nerve injection with each drug was assessed using the pinprick test as previously described (Tochitsky et al., 2021). Briefly, the mice were placed in an individual plastic chamber on a testing rack with a wire mesh floor and habituated for 1 h prior to the peri-sciatic injection. Thereafter, the mice were returned to the testing chamber. An Austerlitz insect pin (size 000) (FST, Vancouver, Canada) was gently and perpendicularly applied to the plantar surface of the paw without penetrating the skin. A response was considered positive if the animal briskly flinched, removed, or licked its paw. The pinprick stimulus was applied five times and the response rate was calculated. The anesthetic effect was considered a complete sensory block if the mice did not respond to any of five pinprick stimuli.

#### Toe Spread Test

After the sciatic nerve block, motor function was assessed using the toe spread test as previously described (Ma et al., 2011). Briefly, the mice were lifted by the tail, uncovering the hind paws for clear observation. Under these conditions, the toe spreading reflex (the spread of the digits to maximize the space between them) was evaluated and scored as follows: no spreading (0, complete motor block), partial toe spreading (1), and full toe spreading (2). Full toe spreading was confirmed in the contralateral paws of all tested mice.

### Experiment 2

#### Zymosan Model of Inflammatory Pain

The analgesic effects of low-dose amiodarone in the inflamed tissues were assessed using the zymosan model of inflammatory pain in another set of 16 mice (Randich et al., 1997). To induce local inflammation and inflammatory pain, the mice were gently restrained in a cloth pocket, and 10 µL of zymosan (5 μg/μL) was injected into the plantar region of the left hind paw using a 30-gauge insulin needle. Consequently, 10 µL of either 0.2% amiodarone or vehicle was injected into the left hind paw (*n* = 8 each; four females and four males). Mechanical and thermal sensitivity and paw thickness were assessed using the von Frey and Hargreaves tests and a digital micrometer at the baseline and at 1, 2, 4, 6, 8, 12, and 24 h after injection. For the mechanical and thermal sensitivity assessments, all mice were acclimatized to each testing environment for 1 h during the 3 days prior to the experiment. After obtaining two baseline values on separate days, inflammatory pain was induced using zymosan injection. Mice were placed on the testing rack for 2 h after zymosan injection for testing at 1 and 2 h. Mice were then placed in their home cage to rest with food and water and returned to the testing rack 30 min prior to each time point (4, 6, 8, 12, and 24 h).

#### Von Frey Test

Mechanical sensitivity was assessed using the von Frey test as previously described (Lee et al., 2019). The mice were placed in a plastic chamber on the same testing rack used for the pinprick test. Responses to mechanical stimuli were measured using a graded series of eight von Frey filaments (bending forces of 0.04, 0.07, 0.16, 0.4, 0.6, 1, 2, and 4 g). Each filament was applied once to the bending point. A positive response was defined as a flinching, licking, or withdrawal of the leg, indicating the mouse has clearly perceived the stimulus. The 50% withdrawal threshold was calculated using the up-down method and the UpDownReader software (Dixon, 1980; Gonzalez-Cano et al., 2018).

#### Hargreaves Test

The thermal nociceptive threshold was assessed using the Hargreaves method. Mice were placed in the same plastic chamber used for the von Frey test on an elevated glass floor preheated at 29–30°C. A radiant heat source (NeoHaroBeam, Toshiba, Tokyo, Japan) was positioned underneath the glass platform, such that the heat was focused on the plantar surface of the paw. Prior to the experiment, the heat intensity was adjusted such that the withdrawal latencies of the naïve paws were between 10 and 15 s. Heat stimulation was repeated three times at an interval of at least 3 min for each paw, and the results were averaged to determine the mean withdrawal latency.

### Experiment 3

#### Systemic Toxicity Assessment

Using another set of 12 mice, the possible cardiovascular and neurological side effects of local amiodarone were assessed by a hemodynamic evaluation and the spontaneous activity test, respectively. Mice received either 1% amiodarone or vehicle by a bolus subcutaneous injection in the dorsum (three females and three males per each group). The amiodarone dose used in this experiment (1 mg per mouse) was five times higher than that used in the sciatic nerve block model (Experiment 1) and 50 times higher than that used in the inflammatory pain model (Experiment 2). Hemodynamic and neurological assessments were performed at the baseline and at 30 min, 1, 2, 4, 8, and 12 h after injection.

#### Hemodynamic Measurement

The possible effects of local amiodarone administration on hemodynamic parameters (heart rate, heart rhythm, and arterial blood pressure) were evaluated. Under gentle restraint using a cloth pocket, a three-probe electrocardiogram was performed to measure heart rate and to assess heart rhythm (PowerLab, Bioamp, and LabChart 8, AD Instruments, NSW, Australia). Arterial blood pressure was measured noninvasively using a tail-cuff method (Softron, Tokyo, Japan).

#### Spontaneous Activity Test

The open-field test was used to detect hyper- and hypoactivity after amiodarone administration and to perform an overall observation of the mice (Rodrigues et al., 2002; Yin et al., 2016). A 50 × 50 × 50-cm observation chamber with a 5 × 5 grid on the floor (25 squares) was used for this test. Prior to the experiment, the mice were acclimatized to the testing environment for 1 h on two consecutive days. The number of lines crossed with all paws during a 5-min period was counted by an individual blinded to the treatment. Another blinded observer performed an overall assessment of the animal’s behavior to detect any locomotor and neurological abnormalities (sedation, leaning, agitation, shivering, and convulsion) during the test period.

### Statistical Analysis

Statistical analysis was performed using the Prism 9 software (GraphPad, San Diego, CA, United States). Pinprick and toe spread scores were analyzed by repeated measures two-way analysis of variance (ANOVA) followed by Tukey’s multiple comparisons test. The duration of complete sensory and motor blockade was analyzed using one-way ANOVA followed by Tukey’s multiple comparisons test. Mechanical withdrawal threshold, thermal withdrawal latency, hemodynamic parameters, and open-field score were analyzed using repeated measures two-way ANOVA followed by the Sidak multiple comparisons test (vs. vehicle). Experimental sample size was determined to detect a difference in the responses of 15% while providing 80% power with an *α* level of 0.05 (G*Power 3.1.9.3). Data were presented as mean ± SEM. Statistical significance was set at *p* < 0.05.

## Results

**FIGURE 1:**
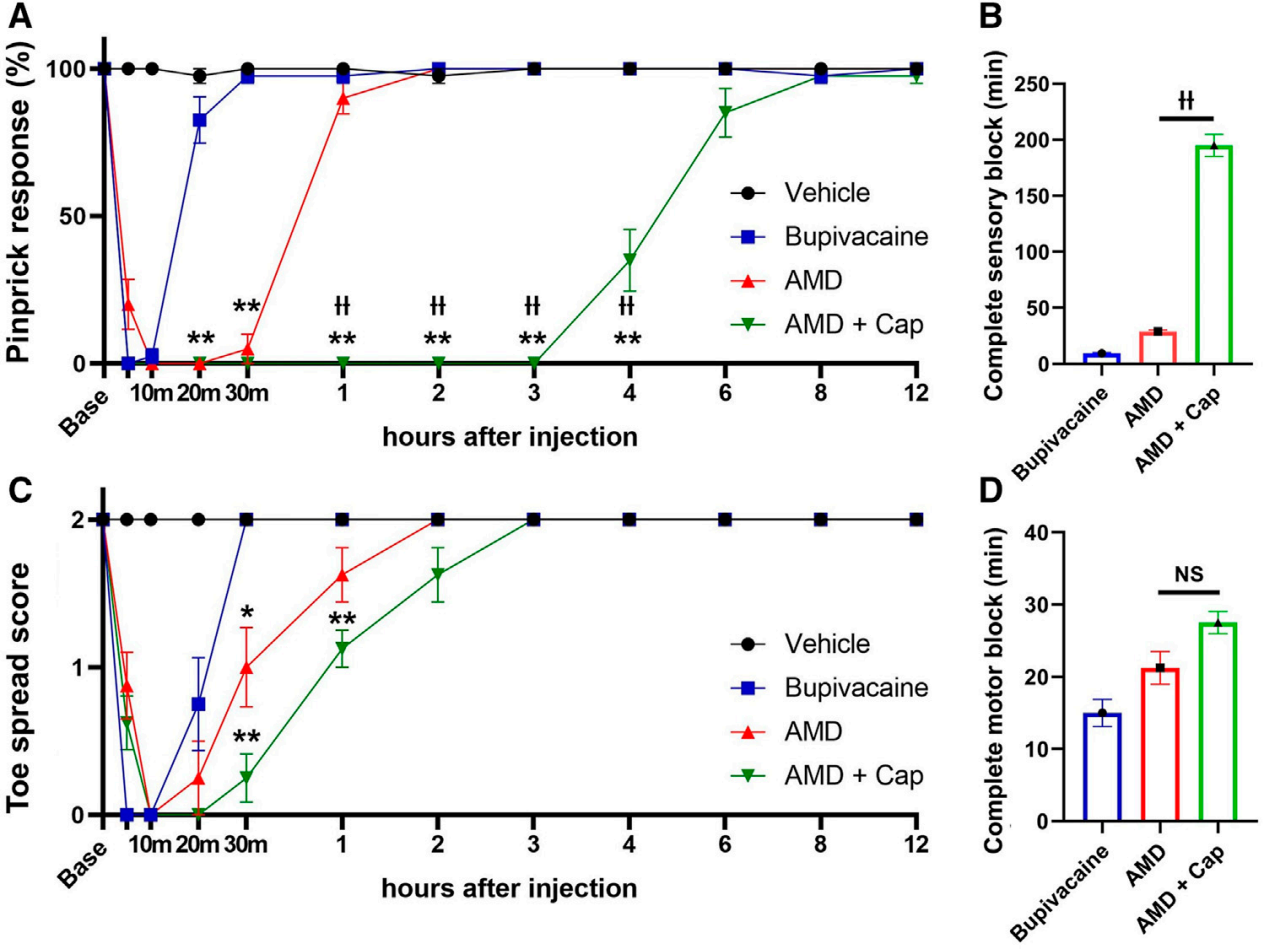
Sensory and motor blockade after peri-sciatic nerve injection using the pinprick and toe spread tests (*n* = 8 each). **(A)** Pinprick response rate. **(B)** Duration of complete sensory block defined as the pinprick response rate of 0%. **(C)** Toe spread score. **(D)** Duration of complete motor block defined as the toe spread score of 0. AMD: amiodarone; Cap: capsaicin *p < 0.05 vs. bupivacaine; **p < 0.01 vs. bupivacaine ††*p* < 0.01 vs. AMD.

In the peri-sciatic injection model, amiodarone produced significantly longer-lasting sensory and motor blockade, compared with bupivacaine (pinprick response at 30 min: 5.0% ± 5.0% vs. 97.5% ± 2.5%, *p* < 0.01; toe spread score at 30 min: 1.0 ± 0.27 vs. 2.0 ± 0.0, *p* < 0.01; Figure 1). Capsaicin coadministration significantly prolonged the duration of the sensory blockade by amiodarone but not that of the motor blockade [complete sensory block duration: 195.0 ± 9.8 min vs. 28.8 ± 1.3 min, F (2, 21) = 317.6, *p* < 0.01, complete motor block duration: 27.5 ± 1.6 min vs. 21.3 ± 2.3 min, F (2, 22) = 11.1, *p* = 0.070].

**FIGURE 2:**
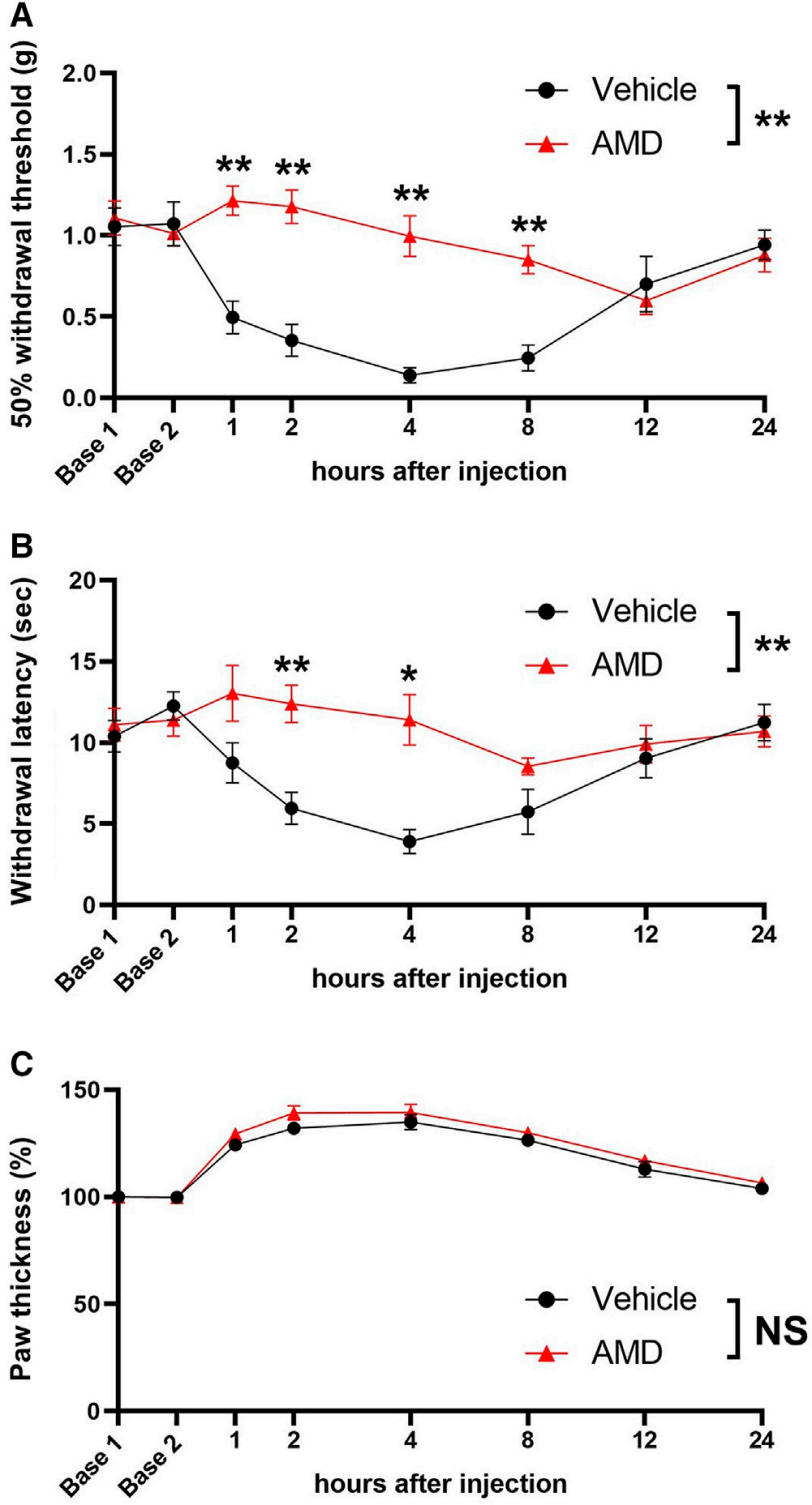
Mechanical and thermal sensitivity and paw thickness after zymosan injection. **(A)** 50% withdrawal threshold values based on the von Frey test with the up-down method. **(B)** Thermal withdrawal latency assessed by the Hargreaves test. **(C)** Paw thickness after zymosan injection AMD: amiodarone *p < 0.05 vs. vehicle; **p < 0.01 vs. vehicle.

In the inflammatory pain model, zymosan produced transient mechanical and thermal hypersensitivity in the vehicle-treated mice, which was reversed by amiodarone for up to 8 h [50% withdrawal threshold at 8 h (g): 0.85 ± 0.09 vs. 0.25 ± 0.08, *p* < 0.01, withdrawal latency at 4 h (s) 8.5 ± 0.5 vs. 5.7 ± 1.4, *p* < 0.05; Figures 2A,B]. The paw thickness of the amiodarone group mice was slightly larger than that of the vehicle group mice, but the difference was not statistically significant [F (1, 14) = 2.50, *p* = 0.136] (Figure 2C).

**FIGURE 3:**
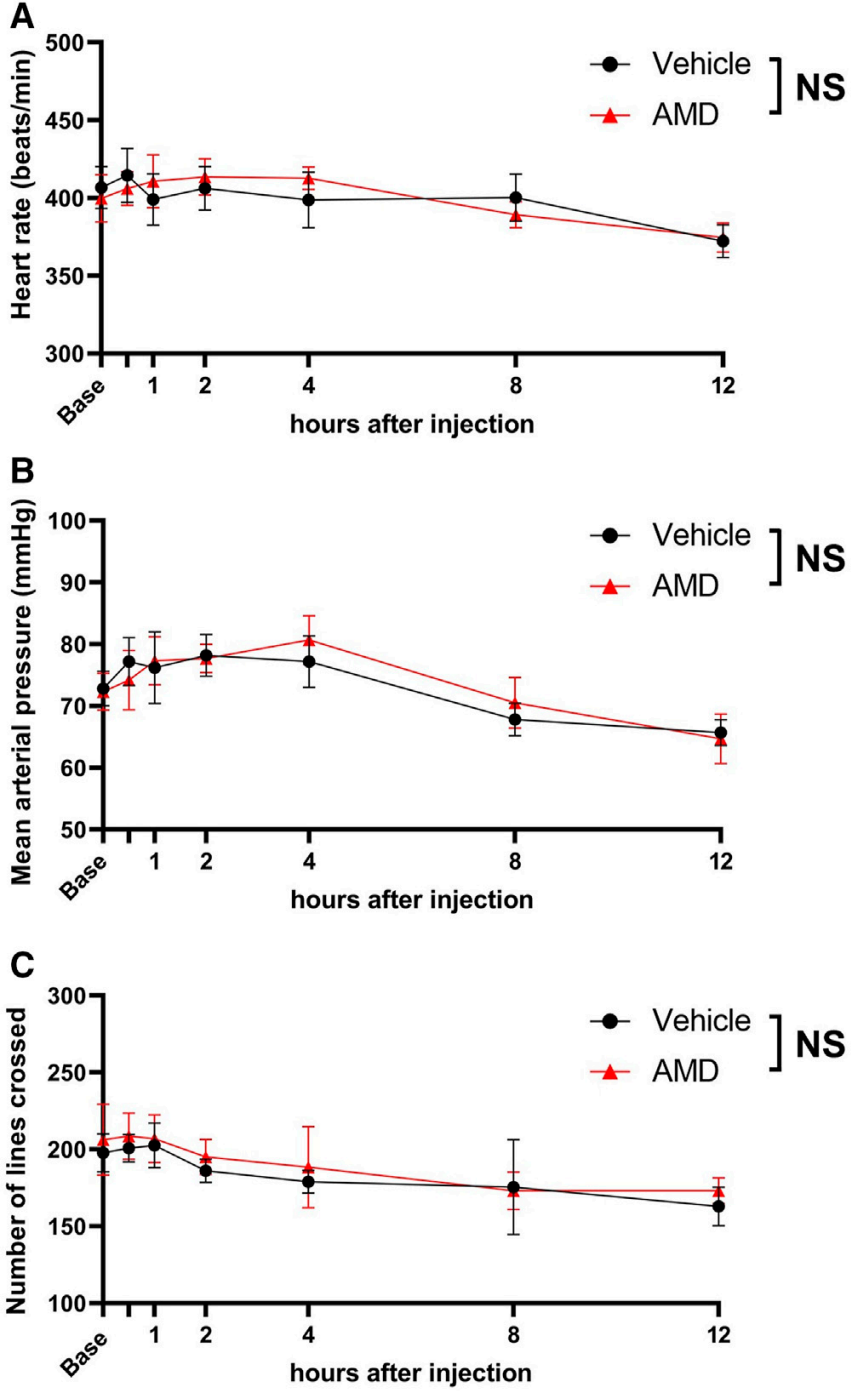
Assessment of cardiovascular and neurological effects by local amiodarone. **(A)** Heart rate measured using three-probe electrogram. **(B)** Noninvasive mean arterial blood pressure measured using the tail-cuff method. **(C)** Spontaneous activity after zymosan injection assessed using the open-field test. The number of lines crossed with all paws during a 5-min period was counted. AMD: amiodarone.

As shown in Figures 3A,B, there were no significant differences in the heart rate and mean arterial blood pressure between the amiodarone and vehicle groups [heart rate: F (1, 10) = 0.01, *p* = 0.909; blood pressure: F (1, 10) = 0.02, *p* = 0.905]. All mice showed a regular sinus rhythm at all time points and did not present with arrythmia. In the open-field test, no significant difference in the spontaneous activity was found between the amiodarone and vehicle groups [F (1, 10) = 0.31, *p* = 0.590]. All mice walked and behaved normally without any neurological symptoms indicating central nervous system side effect.

## Discussion

In the present study, we found that amiodarone produced long-lasting anesthesia in a mouse model of sciatic nerve block and that co-administration of the TRPV1 agonist capsaicin selectively prolonged the duration of the sensory blockade. Moreover, amiodarone produced long-lasting analgesic effects against zymosan-induced inflammatory pain, without the need of a TRPV1 agonist.

Local anesthetics have been used in various clinical settings. These drugs exert its anesthetic effects mainly by blocking voltage-gated sodium channels (Fozzard et al., 2011). Although amiodarone is not a typical sodium channel blocker, it blocks voltage-gated sodium channels by inhibiting fast inward sodium currents through the cellular membrane. The inhibitory effects are both use- and dose-dependent and resemble the pharmacological characteristics of lidocaine (Kodama et al., 1997). We have previously reported that systemic amiodarone exerts analgesic effects by inhibiting sodium channels (Kotoda et al., 2019). Datta et al. also demonstrated that amiodarone attenuates hyperalgesia in an experimental model of neuropathic pain, possibly by blocking sodium channels (Datta et al., 2004). The present results are in line with these findings and demonstrate that amiodarone provides long-lasting local anesthesia, as well as analgesic effects against inflammatory pain.

Generally, the duration of local anesthesia is associated with the degree of protein binding (Covino, 1986; Taylor and McLeod, 2020). Lipid solubility primarily determines the compound potency as a local anesthetic (Covino, 1986; Taylor and McLeod, 2020), but also affects anesthesia duration (Butterworth et al., 2013). Considering that amiodarone has lidocaine-like activity on voltage-gated sodium channels with a high protein binding ratio and partition coefficient, it is not surprising that amiodarone produced long-lasting local anesthetic effects in the present study. The pKa of amiodarone is 8.7 (Chatelain et al., 1986), and this drug exists mostly in its cationic charged form in the tissue. Typically, charged molecules cannot permeate the cellular membrane. However, amiodarone, even in its charged form, may be able to pass through the membrane due to its high liposolubility. Capsaicin activates large-pore TRPV1 channels, creating numerous “large holes” selectively in the TRPV1-expressing sensory neurons. These holes would accelerate the entrance of charged amiodarone molecules directly into the sensory neurons, which could explain the mechanism underlying the enhanced sensory anesthesia observed in the amiodarone + capsaicin group in the sciatic nerve block model.

Subsequently, we examined whether amiodarone alone provides long-lasting analgesia in inflamed tissues, where large-pore channels are already activated. Previous studies have demonstrated that inflammation is associated with activated large-pore channels including TRPV1 (Bujak et al., 2019). We used a widely used mouse model of inflammatory pain, in which subcutaneous zymosan injection induced inflammation and mechanical and thermal hypersensitivity. In this model, we found that low-dose amiodarone alone was effective in reversing the mechanical and thermal hypersensitivity induced by zymosan for up to 8 h, supporting our research hypothesis.

In the clinical setting, amiodarone is intravenously administered at a dose of 300 mg to treat intractable arrhythmia in adult humans. Based on BSA, this dose is equivalent to approximately 1.5 mg for a 25 g adult mice (Baker et al., 2002; Cheung et al., 2009). The present study used amiodarone doses of 0.2 mg for the peri-sciatic injection and 0.02 mg for the intraplantar injection, each of which is considered safe and should not cause systemic side effects. Herein, we confirmed that 1 mg of subcutaneous amiodarone did not cause cardiovascular or neurological side effects.

This study has several limitations. Although capsaicin seems pharmacologically effective in rendering cationic compounds able to selectively enter sensory neurons, capsaicin administration and consequent TRPV1 channel activation produce pain sensation; therefore, local capsaicin injection is not realistic in clinical settings. Conversely, our results show that injecting amiodarone alone into the inflamed tissue may be a more practical method to take advantage of the long-acting anesthetic/analgesic effects of amiodarone, as large-pore channels are already activated, and capsaicin coadministration is not required. Another limitation is that the cellular membrane has been considered the primary barrier for local anesthetic diffusion, and we thoroughly discussed the condition of the membrane in this study. However, a recent computer simulation study indicated the possibility that the cellular membrane is not a significant barrier, and the diffusion of local anesthetics depends more on the pH of the tissue. In addition, we did not collect information regarding the estrous cycle of the mice, which could have influenced pain perception. Lastly, local anesthetics are neurotoxic in high concentrations or with prolonged duration (Markova et al., 2020), and amiodarone could also be neurotoxic. Amiodarone is highly liposoluble and could concentrate in membranes or myelin sheets of the neurons. Although mice that received amiodarone showed full functional recovery after 24 h in the sciatic nerve block and intraplantar injection models, the possibility of potential nerve damage caused by amiodarone cannot be excluded. In addition, amiodarone is known to have tissue-irritating effects. Therefore, limiting its use to low doses may be important. Our future studies will seek to identify amiodarone-variant drugs with long-acting anesthetic and analgesic profiles but without neurotoxicity and tissue-irritating effects.

## Conclusion

Amiodarone produced robust anesthesia in the sciatic nerve block model and co-administration of capsaicin, a TRPV1 agonist, selectively prolonged the duration of the sensory blockade. Low-dose amiodarone was effective against zymosan-induced inflammatory pain, reversing the mechanical and thermal hypersensitivity not requiring capsaicin co-administration. These results indicate that amiodarone may be a long-acting and nociceptor-selective local anesthetic and analgesic method targeting open-state large-pore channels.

## Data Availability

The raw data supporting the conclusion of this article will be made available by the authors, without undue reservation.
